# Gut Microbiota Dysbiosis Accelerates Prostate Cancer Progression Through Increased LPCAT1 Expression and Enhanced DNA Repair Pathways

**DOI:** 10.3389/fonc.2021.679712

**Published:** 2021-06-17

**Authors:** Yufei Liu, Chen Yang, Zheyu Zhang, Haowen Jiang

**Affiliations:** Department of Urology, Huashan Hospital, Fudan University, Shanghai, China

**Keywords:** microbiota dysbiosis, *Ruminococcus*, glycerophospholipid, LPCAT1, DNA repair

## Abstract

Gut microbiota dysbiosis is related to cancer development and progression. Our previous study showed that *Ruminococcus* was more abundant in CRPC (Castration-resistant prostate cancer) than HSPC (Hormone-sensitive prostate cancer) individuals. Here, we determined the potential mechanism of microbiota dysbiosis in prostate cancer (PCa) progression. Metagenomics was used to verify the gut microbial discrepancies between CRPC and HSPC individuals. Fecal microbiota transplantation (FMT) was performed by transferring the fecal suspension of CRPC or HSPC individuals to TRAMP mice. Afterwards, the mice’s prostate histopathology and gut microbiota composition were determined. Since *Ruminococcus* was demonstrated to correlate with phospholipid metabolism, we used lipidomics to examine the mice’s fecal lipid profiles. The expression of LPCAT1 the key enzyme for phospholipid remodeling in mice prostate was also examined. Meanwhile, both microbial functions prediction and LPCAT1 GSEA analysis (Gene Set Enrichment Analysis) indicated DNA repair pathways, we further determined the expressions of RAD51 and DNA-PKcs in mice prostate. The results showed that gut *Ruminococcus* was significantly more abundant in CRPC individuals. FMT using CRPC feces accelerated mice’s PCa progression and increased their gut *Ruminococcus* abundance. Majority of fecal lipids including lysophosphatidylcholine and phosphatidylcholine were upregulated in CRPC FMT treated mice, accompanied with enhanced expressions of LPCAT1, RAD51, and DNA-PKcs in mice prostate. We reported an abundant colonization of *Ruminococcus* in the gut of CRPC individuals and mice receiving their fecal suspensions, and revealed the promotive capability of *Ruminococcus* in PCa progression *via* upregulating LPCAT1 and DNA repair protein expressions. The bacterium and its downstream pathways may become the targets of therapies for PCa in the future.

## Introduction

Recent studies have shown that individuals with prostate cancer (PCa) have gut microbiotas different from that of men with benign prostate conditions (1, 2). However, due to relatively small sample sizes and racial differences, the role gut microbiota plays in prostate pathogenesis in Chinese men remains unclear. We previously used 16s rRNA sequencing to find that gut *Ruminococcus* was significantly more abundant in castration-resistant prostate cancer (CRPC) than hormone-sensitive prostate cancer (HSPC) patients (3). Aberrant abundance of *Ruminococcus* was associated with inflammatory bowel disease and colonic neoplasia (4, 5). In a study trying to treat ulcerative colitis patients with fecal microbiota transplantation (FMT), the authors found that patients who received FMT from donors with high *Ruminococcus. gnavus* level were more likely to relapse (6). Investigation of *Ruminococcus* related signaling pathways would help understand the roles that gut microbiota dysbiosis play in PCa progression.

Gut microbiota is tightly associated with material metabolism in humans. Zhang, et al. found in HIV-infected women that *Ruminococcus* was among the top bacterial genera that contribute to glycerophospholipid metabolism and was positively correlated with plasma glycerophospholipid, lysophosphatidylcholine acyl, and phosphatidylcholine acyl levels (7). The involvement of *Ruminococcus* in glycerophospholipid metabolism was also reported in atherosclerotic cardiovascular diseases (8). Lysophosphatidylcholine acyltransferase 1 (LPCAT1) is a key enzyme responsible for phospholipid biosynthesis/remodeling. Upregulation of LPCAT1 has been described in kidney cancer, lung cancer, breast cancer, et al., and often heralds poor prognosis (9–11). Overexpressed LPCAT1 also indicated PCa progression and biochemical recurrence (12, 13).

Differences in microbial function between CRPC and HSPC individuals included activated DNA repair pathways, especially non-homologous end joining (NHEJ) in CRPC cohorts (3). Interestingly, GSEA analysis (Gene Set Enrichment Analysis) showed that LPCAT1 amplification status was positively related to DNA repair signaling pathways (See the Results section). Homologous recombination (HR) and NHEJ are the two major repair methods for DNA double-strand breaks (DSBs). HR is an error-free approach which uses homologous template to repair, but it works merely in cellular S and G2 phases. HR is mediated by RAD51 protein align with factors such as BRCA1 or BRCA2. NHEJ is a more efficient repair method that works in all cell cycle phases. During NHEJ, DSBs are initially recognized by Ku70/80, then appeals to catalytic subunit DNA-PKcs that orchestrates the repair process by phosphorylating itself and ligase IV-XRCC4-XLF complex. Many studies have found overexpressed NHEJ during cancer progression, recurrence, and chemoradiotherapy resistance (14–16). Inhibition of DNA-PKcs induced cancer cell apoptosis and increased the sensitivity to chemoradiotherapy (17, 18).

Based on above mentioned, we proposed the hypothesis that *Ruminococcus* may accelerate PCa progression by upregulating the expression of LPCAT1 and DNA repair proteins. This study utilized FMT experiment, animal model, multi-omics and molecular biology to explore the mechanism of gut microbiota dysbiosis in PCa progression *via* “*Ruminococcus*-LPCAT1-DNA repair” axis.

## Materials and Methods

### Human Subjects

This study was approved by IRB of Huashan hospital Fudan University (Grant No.2020-531). Clinical database of our department between March 2016 and November 2019 were searched. Written informed consent were obtained from participants. Participants meet the following criteria were recruited: 1) Diagnosis of PCa by histopathology; 2) Receive ADT after initial diagnosis-with or without radical prostatectomy; 3) Progression to CRPC. But Individuals were excluded if: 1) take antibiotics within 30 days of enrollment; 2) have a history of gastrointestinal diseases; 3) receive other forms of systemic chemoradiotherapy before enrollment. 21 participants were eventually enrolled, paired feces were collected at initial diagnosis before ADT, and at CRPC after ADT (3). Fecal samples were collected by patients using a sterile “feces tube” with screw cap (often several times). The tube contains a spoon that is fixed to the cap and projects down the tube. Fresh feces were immediately subpackaged and stored frozen at –20°C after production and then were transferred to –80°C until used. The diagnostic criteria for CRPC was complied with EAU guidelines on prostate cancer (19): Castrate serum levels of testosterone (testosterone <50 ng/dl or <1.7 nmol/l); Three consecutive rises of prostate-specific antigen (PSA), 1 wk apart, resulting in two 50% increases over the nadir with PSA >2.0 ng/ml; Antiandrogen withdrawal for at least 4 wk for flutamide and for at least 6 wk for bicalutamide; PSA progression, despite consecutive hormonal manipulations; Progression of osseous lesions: progression or appearance of two or more lesions on bone scan or soft tissue lesions using Response Evaluation Criteria in Solid Tumours and with nodes >2 cm in diameter.

### Shotgun Metagenomic Sequencing and Analysis

Five patients were randomly selected and their paired feces (HSPC or CRPC) were examined. The patients’ clinical information was displayed (**Table 1**). Total microbial genomic DNA was extracted with DNeasy PowerSoil Kit (QIAGEN, Inc., Netherlands) according to manufacturer’s instructions, and stored at -20°C. The quantity and quality of DNAs were measured using a NanoDrop ND-1000 spectrophotometer (Thermo Fisher Scientific, MA, USA) and agarose gel electrophoresis. Extracted DNA was processed to construct metagenome shotgun sequencing libraries with Illumina TruSeq Nano DNA LT Library Preparation Kit, and sequenced with Illumina HiSeq X-ten platform using PE150 strategy (Shanghai Personal Biotechnology Co., Ltd., Shanghai, China).

**Table 1:** Clinical characteristics of patients.

Case	Age(y)	GS	PSA (ng/ml)	Race	BMI	Methods of ADT	Time to CRPC (Month)	Metastatic
1	69	5+4	9.08	Yellow	19.9	LHRHa+antiandrogen	8	Y
2	68	4+5	38.2	Yellow	21.4	LHRHa+antiandrogen	12	Y
3	67	4+5	420.6	Yellow	24.8	LHRHa+antiandrogen	21	Y
4	61	4+5	100	Yellow	18.5	LHRHa+antiandrogen	9	Y
5	69	5+5	398.8	Yellow	21.7	LHRHa+antiandrogen	14	Y

GS, Gleason score; ADT, androgen deprivation therapy; LHRHa, Luteinizing hormone-releasing hormone agonist; CRPC, castration-resistant prostate cancer.

Sequencing adapters were discarded (20), low-quality reads were trimmed, then reads were filtered to eliminate human host contamination (21). Quality-filtered reads were assembled to construct the metagenome of each sample by IDBA-UD (22). All coding regions of metagenomic scaffolds >300 bp were predicted using MetaGeneMark, and coding region sequences of all samples were clustered by CD-HIT to obtain non-redundant gene catalog (23). Gene abundance of each sample was estimated by soap.coverage according to the number of aligned reads. The lowest common ancestor taxonomy of non-redundant genes was obtained by aligning them against the NCBI-NT database by BLASTN (e-value <0.001). Alpha diversity was performed to investigate the richness and compositional variation of microbial communities across groups. LEfSe analysis was performed to detect differential microbiotas with both statistical significance and biological relevance (24). Functional profiles of microbiotas were obtained by annotating non-redundant genes against KEGG database (25).

### Animal Model and FMT Experiment

The animal study was approved by IRB of Fudan University (Grant No. 2020-JS252). TRAMP mice (Jackson Laboratory, Bar Harbor) were bred in Institute of Development Biology and Molecular Medicine of Fudan University. TRAMP male mice (6-8 weeks) were randomly categorized into two groups, singly housed, and maintained in a standard 12-hour light/dark condition. Mice were treated with antibiotics through drinking water containing 0.2 g/L ampicillin, neomycin, and metronidazole, and 0.1 g/L vancomycin daily for one week before human FMT experiment. After one week, mice were orally gavaged with either CRPC or HSPC fecal suspension twice weekly for 12 weeks. After that, the mice were killed, their prostate, blood and stool samples were collected.

The prepare FMT, paired feces (either CRPC or HSPC) from the five selected patients were unfreezed and mixed with saline solution (20 mg ml^-1^) respectively at equal weights, vortexed and centrifuged. An aliquot of 200μl of fecal suspension was administered per time to mouse by oral gavage.

### 16s rRNA Sequencing and Analysis

After FMT, the stool DNA of two groups of mice were extracted respectively using the DNeasy PowerSoil Kit. Regions V3–V4 of the 16S rRNA gene were amplified using the forward primer 5′-ACTCCTACGGGAGGCAGCA-3′, and the reverse primer 5′-GGACTACHVGGGTWTCTAAT-3′. The PCR program was set: 98°C 10min, 25 cycles of 98°C 15 s, 55°C 30 s, 72°C 30 s, and 72°C 5 min. An equimolar amplicon pool was obtained, and paired-end 2×300 bp sequencing was performed using the Illlumina MiSeq platform with MiSeq Reagent Kit v3 (Shanghai Personal Biotechnology Co., Ltd., Shanghai, China).

Raw reads with exact matches to the barcodes were identified as valid sequences. Sequences were discarded if they (a) were <150 nucleotides in length, (b) had average Phred scores of <20, (c) contained ambiguous bases or mononucleotide repeats of >8 bp. Paired-end reads were aligned using FLASH, and delineation of operational taxonomic units (OTUs) was conducted with UCLUST at a 97% cutoff. LEfSe analysis was performed to detect differential microbiotas between the two groups.

### LC-MS/MS Lipid Analysis

The stool lipids of two groups of mice were extracted respectively using the MTBE method (26). Briefly, samples were homogenized with 200 μl 4°C water, flash freezed with liquid nitrogen, then were mixed with 200 μl ice cold methanol and 800 μL MTBE. The mixture was under ultrasound 20 min at 4°C, then was centrifuged 15min at 14000g 10°C. The upper organic solvent layer was gathered and dried with nitrogen.

Lipid analysis was performed as instructions of Shanghai Applied Protein Technology. A UHPLC Nexera LC-30A system was used. Reverse phase chromatography was selected for LC separation using CSH C18 column (1.7 μm, 2.1 mm×100 mm, Waters). The parameters were: column temperature 45°C; flow rate 0.3 mL/min; sample volume 5 μl. Solvent A: acetonitrile–water (6:4, v/v) with 0.1% formic acid and 0.1Mm ammonium formate; Solvent B: acetonitrile–isopropanol (1:9, v/v) with 0.1% formic acid and 0.1Mm ammonium formate. The mobile phase was 30% B from 0-2 min, linearly increased to 100% B from 2-25 min, 30% B from 25-30 min.

Mass spectra was acquired by Q-Exactive Plus in positive and negative mode, respectively. ESI parameters were: Positive: Heater Temp 300°C, Sheath Gas Flow rate 45 arb, Aux Gas Flow Rate15 arb, Sweep Gas Flow Rate 1arb, spray voltage 3.0KV, Capillary Temp 350°C, S-Lens RF Level 50%, MS1 scan ranges: 200-1800. Negative: Heater Temp 300°C, Sheath Gas Flow rate 45arb, Aux Gas Flow Rate 15arb, Sweep Gas Flow Rate 1arb, spray voltage 2.5KV, Capillary Temp 350°C, S-Lens RF Level 60%, MS1 scan ranges: 250-1800. Lipid Search software version 4.1 (Thermo Scientific™) was used for lipid identification, selection and quantification.

### Prostate Histopathology and Immunohistochemistry

The mice prostate was sectioned and fixed in 4% paraformaldehyde, placed in 70% ethanol, dehydrated and embedded in paraffin. The sections were stained with hematoxylin and eosin. The results were blindly given by two independent skilled uropathologists at Huashan hospital based on a modified grading system for prostatic lesions in TRAMP mice (27).

Immunohistochemistry was performed using LPCAT1 antibody (1:200; 66044-1-Ig; Proteintech Group, Inc., Chicago, IL). After washing 3 times with PBS, the sections were incubated with HRP-coupled secondary antibodies for 1h at room temperature. DAB was added for 3-4 min, then the slides were dehydrated with increasing ethanol concentrations and xylene, and sealed.

### Cell Culture and Transfection

RWPE-1 and PCa cell lines (PC3, DU145, LnCaP) were obtained from the Type Culture Collection of the Chinese Academy of Sciences (Shanghai, China) and cultured at 37°C in 5% CO2 using RPMI 1640 medium (Gibco, Gaithersburg, MD, USA) containing 10% FBS (Gibco), 100 U/mL penicillin and 100 μg/mL streptomycin. For cell transfection, LPCAT1 siRNA (siLPCAT1, sc-91777, Santa Cruz Biotechnology, Shanghai, China) or control siRNA (sc-37007, Santa Cruz Biotechnology) was transfected into PC-3 cells *via* Lipofectamine^®^ 3000 (Invitrogen, Carlsbad, CA, USA) according to manufacturer’s protocol. Culture supernatant was harvested after 48 hours, puromycin was used to select transduced cells.

### Cloning Formation Assay and Migration Assay

For colony formation assay, transfected cells were seeded into six-well plates at a density of 500 cells/well and maintained in RPMI 1640 medium containing 10% FBS for 7 days. The colonies were imaged and counted after they were fixed and stained. Cell migration was measured using 24-well Transwell chambers (BD Biosciences, Franklin Lakes, NJ, USA). A total of 1×10^5^ cells were plated into the upper chamber with 200 μL serum-free medium while the bottom chamber was filled with 500 μL completed medium. After culturing for 24 hours, cells in the bottom chamber were fixed with 4% paraformaldehyde for 30 minutes, then stained with crystal violet for 10 minutes.

### Western Blots

The mice prostate tissues or human prostate cell lines were extracted and concentrated using RIPA lysis buffer containing protease inhibitors (Sigma-Aldrich, St. Louis, MO) and BCA Protein Assay kit (Vigorous Biotechnology, Beijing, Beijing, China), respectively. Equal proteins were resolved using SDS-PAGE before being transferred onto nitrocellulose membranes (Millipore, Madison, WI). Membranes were blocked with 5% BSA in TBST buffer and incubated with following primary antibodies (LPCAT1 antibody (1:200; 66044-1-Ig; Proteintech Group, Inc., Chicago, IL); DNA-PKcs antibody (1:200; ab32566; Abcam); p-DNA-PKcs (S2056) (1:400; ab18192; Abcam), and RAD51 antibody (1:200; PA5-27195; Invitrogen™) at 4°C overnight and HRP-coupled secondary antibodies at room temperature for 1h. GAPDH was used as internal control.

### Statistical Analysis

Data were expressed as mean ± standard deviation (SD) and compared between groups using T-test with GraphPad Prism software (Version 5.0, GraphPad, La Jolla, CA, USA). Mann-Whitney U test was applied for nonparametric data analysis. *P ≤* 0.05 indicated statistically significant difference.

## Results

### *Ruminococcus spp*. Was More Abundant Among CRPC Individuals

**Figure 1:**
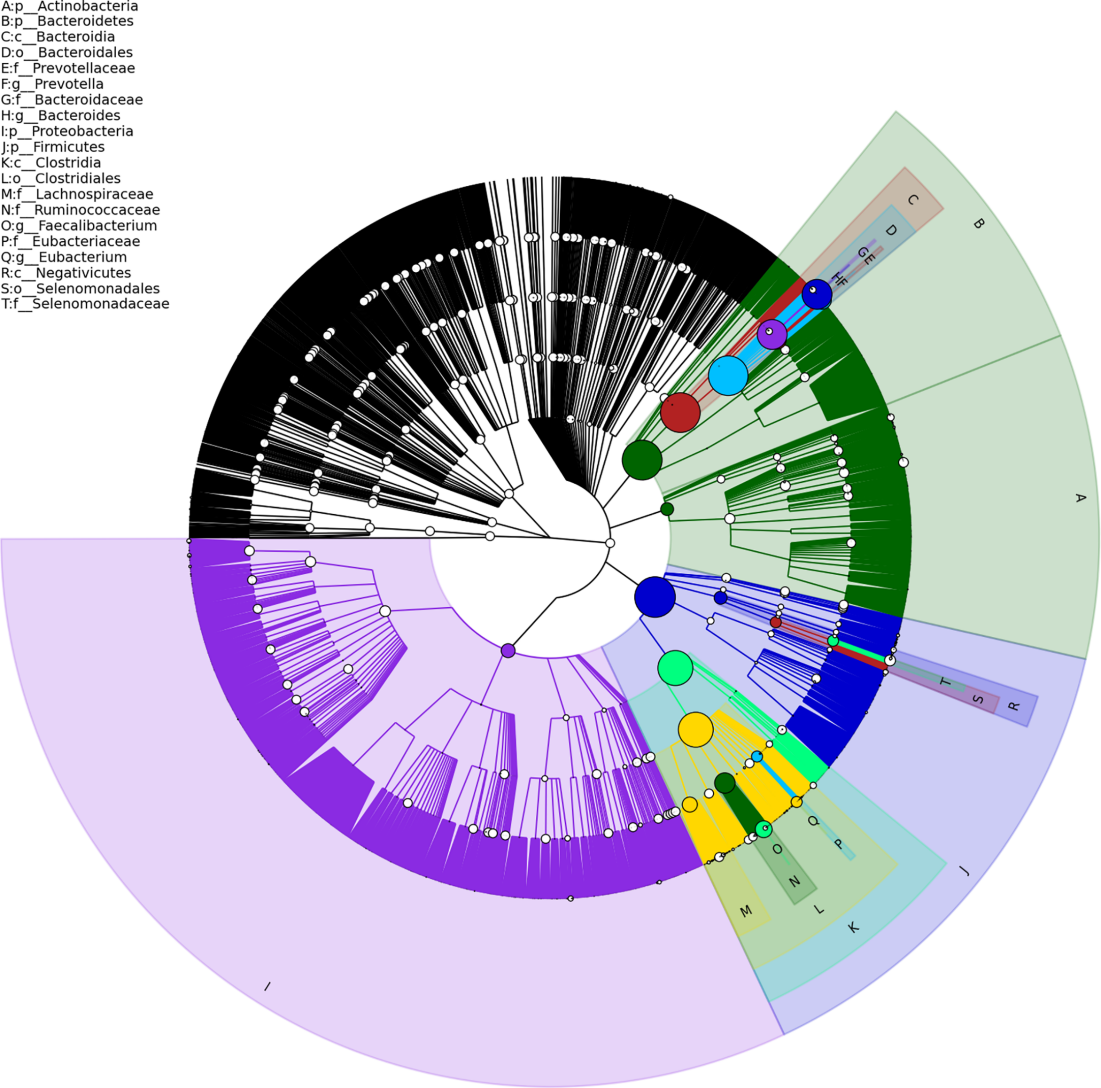
Structure of gut microbial communities in PCa patients. Taxonomic hierarchy tree using GraPhlAn tool displayed the overall structure of gut microbial communities in PCa patients from phylum to species (outer to inner circle). The node size represented the relative abundance of the taxon. The top 20 taxa were annotated.

**Figure 2:**
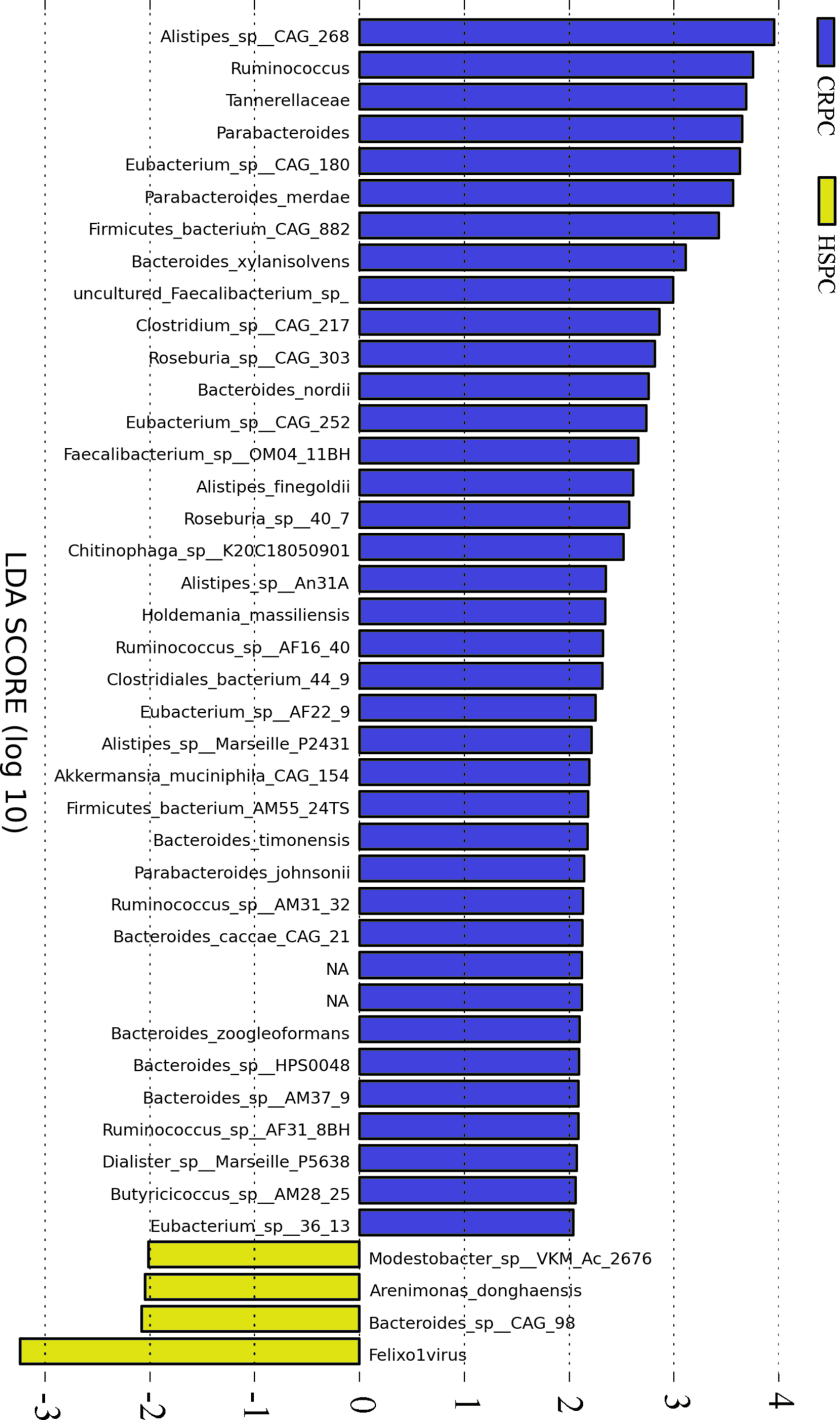
Differentiated microbial phylotypes between CRPC and HSPC individuals. Metagenomics identified 44 differentiated microbial phylotypes between the two cohorts with LEfSe analysis (LDA>2, *P*<0.05), *Ruminococcus* and three subordinate species were more abundant in CRPC individuals.

Gut metagenome detected average 7.33×10^7^ reads of each sample. Taxonomy based on GraPhlAn tool showed that microbial communities in PCa patients were dominated primarily by phylum *Firmicutes (41.63%), Bacteroidetes (40.08%), Proteobacteria (4.90%)* and *Actinobacteria (4.16%)*(**Figure 1**). Chao1 index was used to reflect the richness of microbial communities, the values were 12518.89 ± 2314.92 *vs*.12716.55 ± 2089.95 (*p*=0.89) respectively in CRPC and HSPC individuals indicating that there were no obvious differences in microbiota richness between the two cohort. LEfSe analysis identified 38 differentiated microbiotas, among which 4 were down-regulated and 34 were upregulated in CRPC group. *Ruminococcus* spp. (*Ruminococcus; Ruminococcus_sp_AF16_40; Ruminococcus_sp_AM31_32; Ruminococcus_sp_AF31_8BH*) were significantly more abundant in CRPC individuals (**Figure 2**).

### Accelerated PCa Progression in Mice After FMT With CRPC Feces

**Figure 3:**
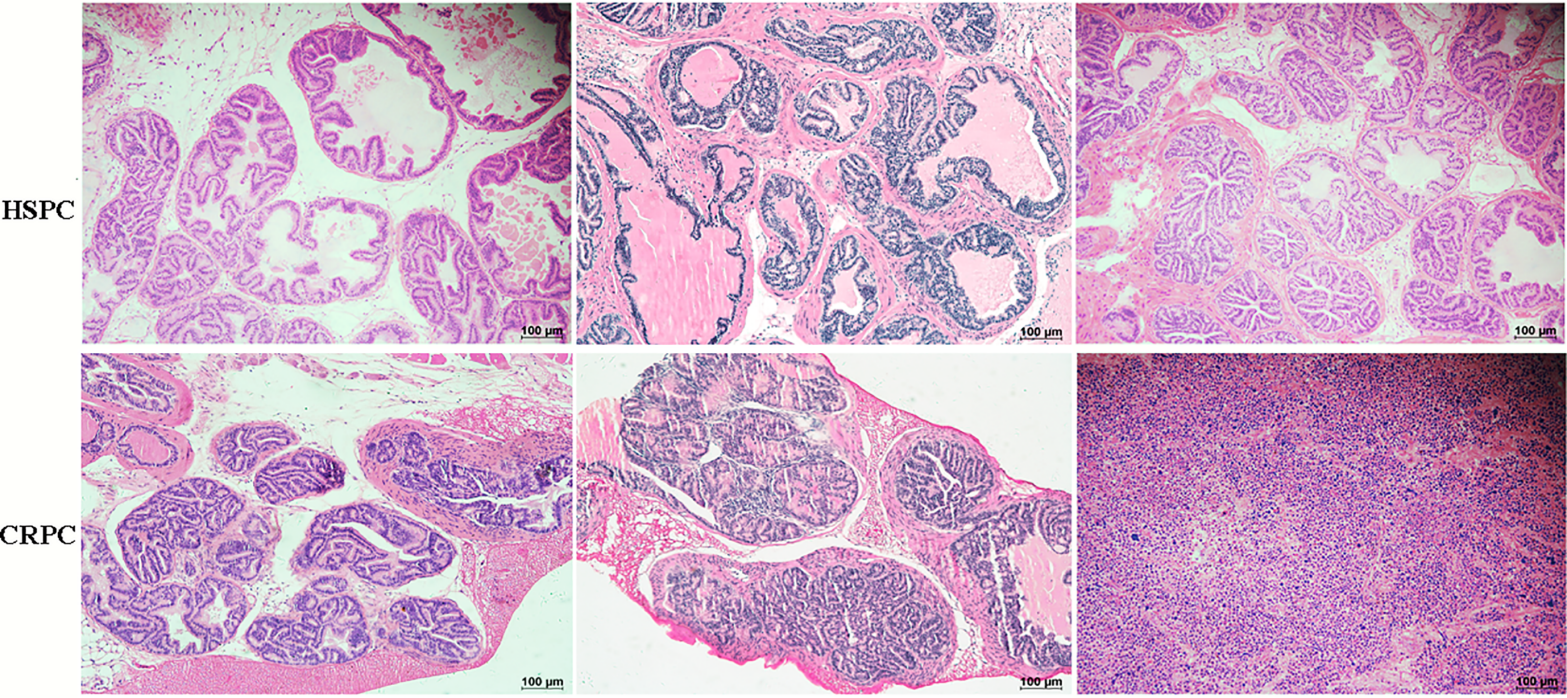
Histopathological images of mice prostate after FMT. FMT with CRPC feces significantly accelerated mice’s PCa progression. The bar represents 100 µm.

To determine the influences of gut microbiota on PCa progression, we conducted FMT experiment to TRAMP mice. After 12 weeks of FMT, mice accepting FMT with CRPC feces exhibited PIN (prostate intraepithelial neoplasia) and PCa (3 high-grade PIN, 3PCa), while their counterparts that received FMT with HSPC feces exhibited only PIN (2 moderate-grade PIN, 4 high-grade PIN) (n= 6 mice per group). FMT with CRPC feces significantly accelerated mice’s PCa progression (*p*=0.030) (**Figure 3**).

### Increased Gut *Ruminococcus* in Mice After FMT With CRPC Feces

**Figure 4:**
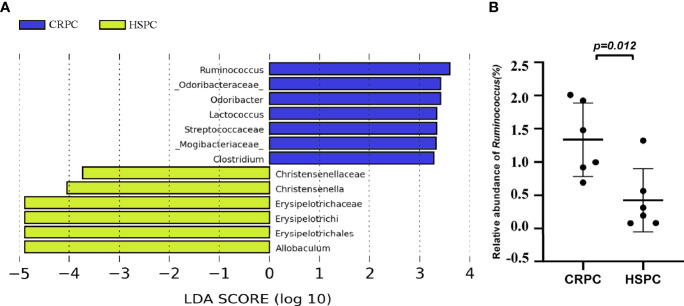
FMT with CRPC feces increased mice’s gut *Ruminococcus*. **(A)** 16s rRNA sequencing identified increased gut *Ruminococcus* in CRPC FMT treated mice. **(B)** Relative abundance of *Ruminococcus* between two groups of mice after FMT. The bars represents mean ± SD.

To further determine the influences of FMT on mice’s gut microbiota, we examined the mice’s gut microbiota after FMT using 16s rRNA sequencing, and found that mice accepting FMT with CRPC feces had higher level of gut *Ruminococcus* (**Figure 4A**). The relative abundance of *Ruminococcus* were 1.34 ± 0.55% vs. 0.43 ± 0.47% (*P*=0.0121), respectively (**Figure 4B**).

### Increased Fecal Lipid Level in Mice After FMT With CRPC Feces

**Figure 5:**
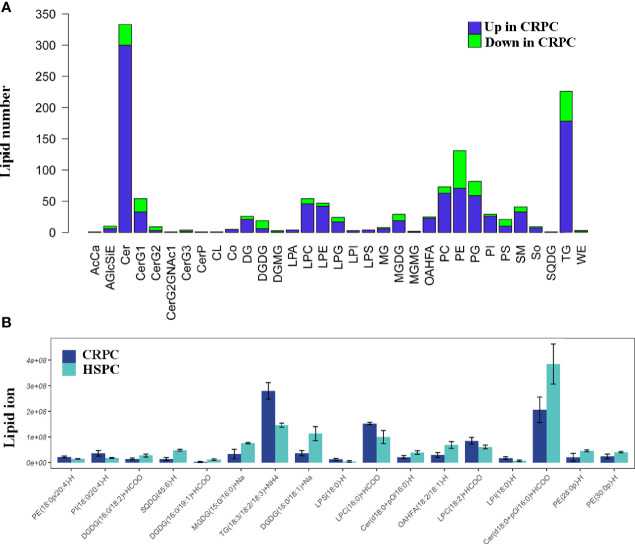
Fecal lipid profiles of mice after FMT. **(A)** Lipidomics showed that the levels of most lipid classes including glycerophospholipids were increased in mice after FMT with CRPC feces. The blue bars indicated the number of lipids within each lipid class that increased in CRPC FMT treated mice., while the greed bars indicated the number of lipids that decreased. **(B)** On the species level, 17 species were significantly different between the two groups, 16:0 LPC and 18:2 LPC were significantly increased in CRPC FMT treated mice.

Since *Ruminococcus* was proved to correlate with glycerophospholipid metabolism, we further examined the fecal lipid contents of mice after FMT. LC-MS/MS identified totally 33 lipid classes and 1283 lipid species. 4 lipid classes including CerG2, DGDG, DGMG, WE were down-regulated, 29 lipid classes including glycerophospholipids (PC, PE, PS, PG, PI, etc) were upregulated in CRPC FMT treated mice (**Figure 5A**). On the species level, 17 species were significantly different between the two groups, LPC(16:0)+HCOO and LPC(18:2)+HCOO were significantly increased in CRPC FMT treated mice (**Figure 5B**).

### Differentiated Microbial Functions and LPCAT1 GSEA Analysis

**Figure 6:**
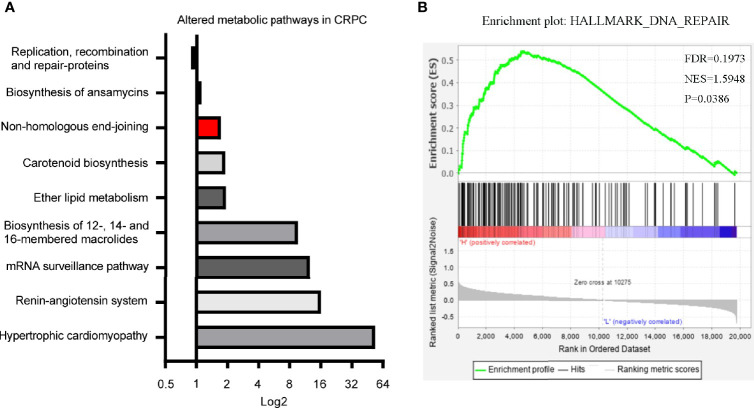
Differentiated microbial functions and LPCAT1 GSEA analysis. **(A)** PICRUSt (Phylogenetic Investigation of Communities by Reconstruction of Unobserved States) identified 11 microbial KEGG pathways that were different between CRPC and HSPC individuals, non-homologous end-joining pathway (Red Bar) was enhanced in CRPC cohort. The bar represented CRPC/HSPC ratio in relative abundance of certain KEGG pathways. **(B)** GSEA analysis of LPCAT1 indicated positive correlation between LPCAT1 amplification status and DNA repair pathways. Cancer Genome Atlas (TCGA) data regarding prostate cancer patients was used. Through comparison between mRNA expression and LPCAT1, patients were separated into LPCAT1 high-expression group and LPCAT1 low-expression group. GSEA (Software GSEA_4.1.0.) was performed to identify enriched signaling pathways associated with LPCAT1 expression, gene sets with *p* value < 0.05, false discovery rate (FDR) < 0.25 were considered significant.

In our previous study, we identified 11 microbial KEGG pathways that showed differences between CRPC and HSPC individuals, including DNA replication and repair (NHEJ), ether lipid metabolism, mRNA surveillance, etc (**Figure 6A**) (3). LPCAT1 is a key enzyme for phospholipid biosynthesis, the GSEA analysis of LPCAT1 showed positive relationship between LPCAT1 amplification status and DNA repair signaling pathways (**Figure 6B**).

### Overexpressed LPCAT1 and DNA Repair Proteins in Mice Prostate After FMT With CRPC Feces

**Figure 7:**
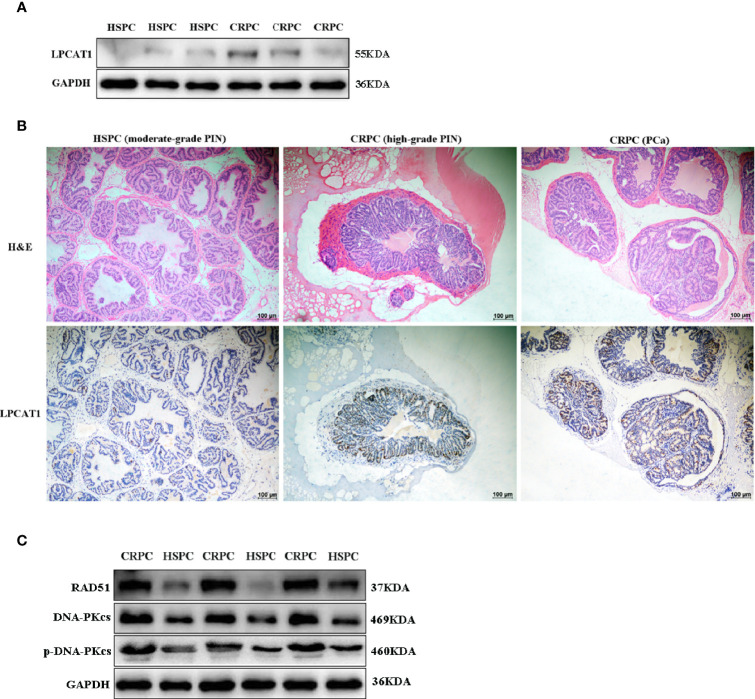
Overexpressed LPCAT1 and DNA repair proteins in mice prostate after FMT with CRPC feces. Both western blots **(A)** and immunohistochemistry **(B)** showed that the expression of LPCAT1 was enhanced in mice prostate after FMT with CRPC feces. **(C)** The expressions of RAD51 and DNA-PKcs were enhanced in mice prostate after FMT with CRPC feces.

The expression of LPCAT1 in mice prostate after FMT was determined. Western blot analysis showed that LPCAT1 was overexpressed in mice accepting FMT with CRPC feces (**Figure 7A**). Immunohistochemistry also showed that LPCAT1 was more intensely stained in CRPC FMT treated mice (**Figure 7B**). Since both microbial function prediction with KEGG database and LPCAT1 GSEA analysis indicated DNA repair signaling pathways, we further determined the expression of DNA-PKcs (NHEJ) and RAD51 (HR) in mice prostate. Western blot analysis showed that DNA-PKcs, p-DNA-PKcs, and RAD51 expression were upregulated in mice accepting FMT with CRPC feces (**Figure 7C**).

### Downregulation of LPCAT1 Suppressed PCa Cell Proliferation and Migration

**Figure 8:**
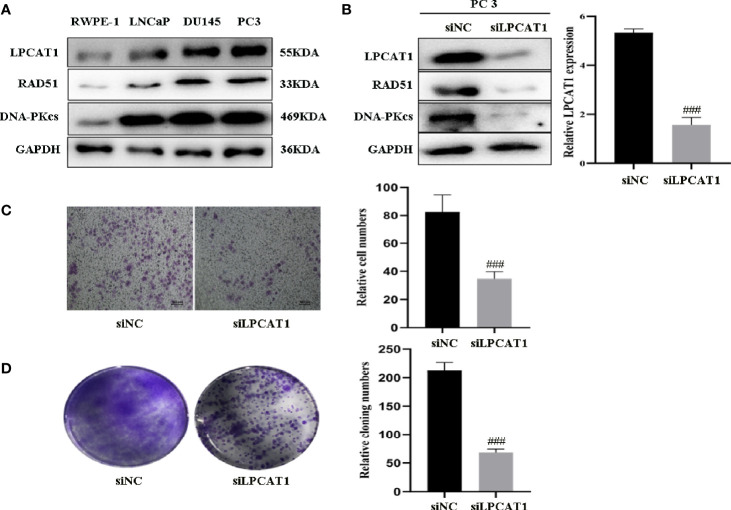
Downregulation of LPCAT1 suppressed PCa cell proliferation and migration **(A)** LPCAT1, DNA-PKcs, RAD51 were highly expressed in PCa cell lines (PC3, DU145, LnCaP) when compared to RWPE-1 (normal prostate epithelial cell). **(B)** Inhibition of LPCAT1 downregulated DNA-PKcs and RAD51 expressions in PC-3 cells. **(C, D)** Cloning formation and migration assays showed that downregulation of LPCAT1 suppressed PC-3 cell proliferation and migration. Data are presented as mean ± SD. ^###^ *P* < .001.

To confirm the relevance of LPCAT1, DNA-PKcs, RAD51 in PCa progression, we characterized their expressions and found that LPCAT1, DNA-PKcs, RAD51 were highly expressed in three PCa cell lines (PC3, DU145, LnCaP) compared to RWPE-1 (normal prostate epithelial cell) (**Figure 8A**). To further confirm that LPCAT1 pathway was involved in PCa progression, PC-3 cells were transfected with LPCAT1 siRNA. Western blot analysis showed that LPCAT1 knockdown suppressed the expressions of DNA-PKcs and RAD51 (**Figure 8B**). Cloning formation and migration assays showed that LPCAT1 knockdown suppressed the proliferation and migration of PC-3 cells (**Figures 8C, D**).

## Discussion

Through integrated analyses of human clinical samples and animal model, we have revealed an essential role of gut *Ruminococcus* in PCa progression. *Ruminococcus* was associated with inflammatory bowel disease and colon neoplasia (4, 5), but its exact role in PCa progression remains elusive. In the present study, we characterized the microbiome associated with CRPC, and determined that FMT with CRPC feces could accelerate mice’s PCa progression, possibly through the activation of “*Ruminococcus*-LPCAT1-DNA repair” pathway.

The results of microbial metagenome complied with our previous study using 16s rRNA sequencing that both indicated higher level of *Ruminococcus* in CRPC individuals. *Ruminococcus* was also reported to be overexpressed in men taking oral androgen receptor axis-targeted therapies in Sfanos’ study (28). *Ruminococcus* is defined as strictly anaerobic, gram-positive, and non-motile cocci. It is one important member of microbial communities both in ruminants and humans, and is disproportionately distributed in certain diseases. Since FMT from CRPC individuals to TRAMP mice resulted in higher abundance of *Ruminococcus*, it seemed that *Ruminococcus* could colonize in intestines. There are merely six named *Ruminococcus* species to date, e.g. the cellulolytic *R. albus* and non-cellulolytic *R. bromii*. Our gut metagenome detected three species belonging to *Ruminococcus* spp. that were increased in CRPC individuals. Since the three were not previous described, their specific functions remain obscure. Here we studied the potential role of *Ruminococcus* spp. in PCa progression.

The involvement of *Ruminococcus* in glycerophospholipid metabolism was reported in several studies. Park T, et al. used FishTaco to identify *Ruminococcus* as the flora potentially contributing to the enriched KEGG pathways for glycerophospholipid biosynthesis (29). In the present study, we examined the lipid profiles in mice feces, and found nearly all glycerophospholipids including lysophosphatidylcholine (LPC) and phosphatidylcholine (PC) were increased by FMT using CRPC feces. Since LPCAT1 participates in LPC/PC metabolism, we further examined the level of LPCAT1 in mice prostate tissues, and found LPCAT1 was overexpressed in CRPC FMT treated mice. Expression of LPCAT1 has been discovered to be elevated in CRPC tissue and its cell line (30), and it allows itself to be used as a prognostic biomarker following prostatectomy (13). However, the exact role of LPCAT1 in PCa progression is unknown. Junfeng B, et al. reported that some growth factor receptor-driven cancers resort to LPCAT1 to remodel the plasma membrane composition through accumulation of phospholipids, which in turn is required for transduction of oncogenic pathways such as EGFRvIII signaling (31). Mansilla, et al. suggested that LPCAT1 in colorectal adenocarcinoma contributed to total choline metabolite accumulation *via* phosphatidylcholine remodeling, thereby altered the lipid profile and increased the malignancy (32), but whether this applies to PCa requires further investigations. Han, et al. reported that LPCAT1 is capable of enhancing CRPC progression *via* increased mRNA synthesis and platelet activating factor production (33). In our study, we proposed a possible explanation about the mechanism of LPCAT1 in PCa progression *via* DNA repair pathways.

We found FMT with CRPC feces upregulated DNA-PKcs and RAD51 expressions in mice prostate tissues. Previous studies have discovered the positive correlation between DNA repair protein expressions and prostate malignancy. For example, application of AR inhibitor Enzalutamide can suppress the expression of HR protein RAD51 in LNCap cells (34). Reduced RAD51 expression was accompanied by reduced fraction of S/G2 phase cells (15). The NHEJ protein DNA-PKcs also interacts with AR (35). Next-generation AR inhibitor Apalutamide can downregulate DNA-PKcs level in both androgen-dependent and CRPC cells (15).

This study is not devoid of limitations, we did not distinguish the causal or casual link between gut *Ruminococcus* and LPCAT1 level in prostate, though we proved that FMT with CRPC feces increased *Ruminococcus* abundance, upregulated prostate LPCAT1 expression. The deeper mechanism requires careful investigations. Study should also be conducted to ascertain and isolate certain specific *Ruminococcus* spp. which is determinative in driving PCa progression. Also, we were unable to trace the change of *Ruminococcus* abundance in CRPC patients, as well as the link between *Ruminococcus* level and clinical outcomes. Finally, our study focused directly on the effects of gut microbiota on PCa. Further study can also examine whether variation of gut microbiota is related to intraprostatic inflammation. Francesca S’ study showed that prostatic inflammation is inversely correlated with presence and aggressiveness of PCa (36). Also, we can examine the microbiota in urine, and explore its correlation with PCa. There is publication reporting that urinary bacterial composition was significantly changed in PCa patients (37), which warranted deeper investigations.

We concluded that FMT with CRPC feces was able to accelerate mice’s PCa progression, and an abundant colonization of *Ruminococcus* had shaped the intestinal microbial structure of CRPC individuals and the mice receiving their fecal suspensions. Our study also revealed the potential capability of *Ruminococcus* to upregulate LPCAT1 and DNA repair proteins expressions. The bacterium and its downstream pathways may become the targets of therapies for PCa in the future.

## Data Availability Statement

The datasets presented in this study can be found in online repositories. The names of the repository/repositories and accession number(s) can be found below: https://www.ncbi.nlm.nih.gov/, PRJNA713539.

## Ethics Statement 

The studies involving human participants were reviewed and approved by IRB of Fudan University. The patients/participants provided their written informed consent to participate in this study. The animal study was reviewed and approved by IRB of Fudan University.

## Author Contributions 

YL and HJ contributed to conception and design of the study. YL organized the database. CY performed the experiments and statistical analysis. YL wrote the first draft of the manuscript. All authors contributed to the article and approved the submitted version.

## Funding

This study was supported by National Natural Science Foundation of China, Grant number: 81872102.

## Conflict of Interest

The authors declare that the research was conducted in the absence of any commercial or financial relationships that could be construed as a potential conflict of interest.
